# Impact of Preanalytical Storage Time and Air Exposure on ROTEM and Coagulation Sample Stability

**DOI:** 10.1155/ah/5145648

**Published:** 2026-02-09

**Authors:** Benjamin S. Storm, Renathe H. Grønli, Kristin Pettersen, Åse E. Emblem, Ingrid S. Joakimsen, Anne Landsem, Tom E. Mollnes, Ole-L. Brekke

**Affiliations:** ^1^ Surgical Clinic, Nordland Hospital Trust, Bodø, Norway; ^2^ Faculty of Nursing and Health Sciences, Nord University, Bodø, Norway, nord.no; ^3^ Department of Clinical Medicine, Faculty of Health, UiT The Arctic University of Norway, Tromsø, Norway, uit.no; ^4^ Research Laboratory, Nordland Hospital Trust, Bodø, Norway; ^5^ Department of Laboratory Medicine, Nordland Hospital Trust, Bodø, Norway; ^6^ Department of Immunology, University of Oslo and Oslo University Hospital, Oslo, Norway, uio.no

**Keywords:** air–blood interface, blood sample, hemostatic measurements, laboratory practice, platelet activation, preanalytical phase

## Abstract

**Introduction:**

The preanalytical phase of blood sample collection, storage, and transportation is crucial in clinical laboratory practice. This study examined the impact of preanalytical storage time and the presence of air in the test tube on viscoelastic and traditional coagulation tests and platelet activation markers.

**Methods:**

Blood samples from ten healthy donors were divided into Vacuette sodium citrate and K2E EDTA tubes, with and without air, and stored at room temperature with gentle agitation for up to 48 h. Coagulation tests (ROTEM, APTT, INR, and PTF1 + 2), platelet activation markers (β‐thromboglobulin and sP‐selectin), blood gases and metabolites (pH, pCO_2_, pO_2_, bicarbonate, hemoglobin, sO_2_, K^+^, Ca^2+^, glucose, and lactate), and hemolysis (free hemoglobin and absorbance) were measured.

**Results:**

In citrated blood samples, ROTEM parameters, APTT, and PTF1 + 2 remained stable during 6 hours of storage at room temperature, and INR was stable for 48 h. In EDTA blood, β‐thromboglobulin increased initially and then remained stable for 6 hours. sP‐selectin remained stable for 6 hours. In EDTA blood samples, hemolysis remained minimal during storage, but cell death revealed by a lactate dehydrogenase increase was observed after 24 h. Blood gases and metabolites exhibited rapid changes, underscoring the need for immediate analysis. No significant differences were observed between samples with and without air in the test tubes.

**Conclusion:**

ROTEM, APTT, PTF1 + 2, and platelet activation markers were stable for 6 h and INR was stable for 48 h. Removing ambient air from blood samples did not extend preanalytical stability. These insights are valuable for optimizing preanalytical practices in clinical laboratories, potentially improving patient care and reducing costs.

## 1. Introduction

The preanalytical phase of blood sampling plays a pivotal role in ensuring the accuracy and reliability of diagnostic results [1]. This phase encompasses a multitude of critical steps, including blood drawing, transportation, storage, and processing of the samples.

Traditionally, blood samples are collected in vacuum tubes that are not absolute vacuums but contain minute amounts of air. These tubes are not fully filled with blood during sampling, resulting in an air–blood interface within the sample tubes. This interface may have implications for the stability and integrity of various analytes within the blood, as air is known to trigger hemostasis and activate complement and platelets in whole blood [2]. To inhibit all biological cascades in blood samples, including coagulation, and facilitate preanalytical sample transportation and storage, sample tubes are supplemented with anticoagulants, such as citrate or ethylenediaminetetraacetic acid (EDTA). Despite the use of these additives, certain analytical tests, such as rotational thromboelastometry (ROTEM), prothrombin time international normalized ratio (INR), and activated partial thromboplastin time (APTT), are known to be time‐sensitive and necessitate swift analysis to yield accurate results [3]. The analytical performance specifications and quality goals of routine analysis from the European Federation of Clinical Chemistry and Laboratory Medicine (EFLM) are currently derived from three different models [4]. These models employ different methodologies for calculating analytical quality goals: Model 1 is based on considerations of clinical outcomes, Model 2 integrates calculations of analytical performance specifications from biological variation data, and Model 3 relies on the application of “state‐of‐the‐art” methods [4, 5]. A key quality goal in Model 2 is the “maximum acceptable bias” calculated from the total biological variation, setting the limit for how much error stemming from factors like sample stability or storage conditions can be tolerated in a measurement [4–6]. The EFLM biological variation database has updated values and meta‐analyses for biological variation data for several analytes.

Delayed analysis or exposure to extended storage and transport periods, particularly at room temperature, can introduce alterations in the composition and characteristics of blood samples, ultimately leading to inaccuracies in diagnostic readings [7–9]. In recent years, viscoelastic coagulation tests, specifically ROTEM and thromboelastography (TEG), have gained popularity. These tests offer a comprehensive assessment of hemostasis, encompassing both plasma coagulation components and platelet function in recalcified citrated whole blood [10, 11]. ROTEM and TEG are often used in a point‐of‐care setup, as storage and exposure to acceleration forces, for example, during sample transport in a pneumatic tube transport system, may activate the hemostatic process and yield unreliable results [12, 13]. Studies on the preanalytical storage of citrated blood before viscoelastic testing have yielded conflicting results. Thus, it is recommended that viscoelastic testing of coagulation using ROTEM is performed within 2 hours of blood collection [14].

The need for accurate and timely analysis of blood samples is particularly relevant for patients on anticoagulation therapy, such as warfarin, where monitoring INR is essential. Many of these patients must travel considerable distances for sample collection. Extending the permissible preanalytical sample storage duration could enable centralized laboratory workflows, allowing for remote sample collection and transportation, reducing the travel costs and patient burden, and facilitating research or field studies where immediate analysis is not possible. Extended sample storage is also practical in hospital settings, as it reduces the need for point‐of‐care analytical instruments and optimizes laboratory logistics. However, extending preanalytical sample storage time may increase inaccuracy and uncertainty, which may be mitigated to some extent through careful sample handling.

We hypothesize that preanalytical sample storage times may be longer than currently prescribed and that mitigating the presence of an air–blood interface in sample tubes may reduce *in vitro* activation of hemostatic processes, including coagulation and platelet activation. In this study, we aim to shed light on the dynamic interplay between air, time, and blood samples in the preanalytical phase. Our primary objective was to investigate the influence of preanalytical sample storage time and the presence of air within sample tubes on a range of analytical parameters, including coagulation, platelet activation, and hemolysis. Our secondary objective was to analyze cell death and to compare hemolysis assessment by two spectrophotometric methods and one clinical chemistry–based method.

## 2. Methods

### 2.1. Blood Sampling

Venous blood was drawn from ten healthy human donors (five men and five women, mean age 42 years, range 28–48 years) onto Vacuette sodium citrate coagulation tubes (Greiner Bio‐One, Kremsmünster, Austria) and Vacuette K2E EDTA tubes (Greiner Bio‐One) using a 21‐G Vacuette Safety Blood Collection set with 19‐cm polyvinyl chloride (PVC) tubing (Greiner Bio‐One) using standard laboratory technique.

**FIGURE 1:**
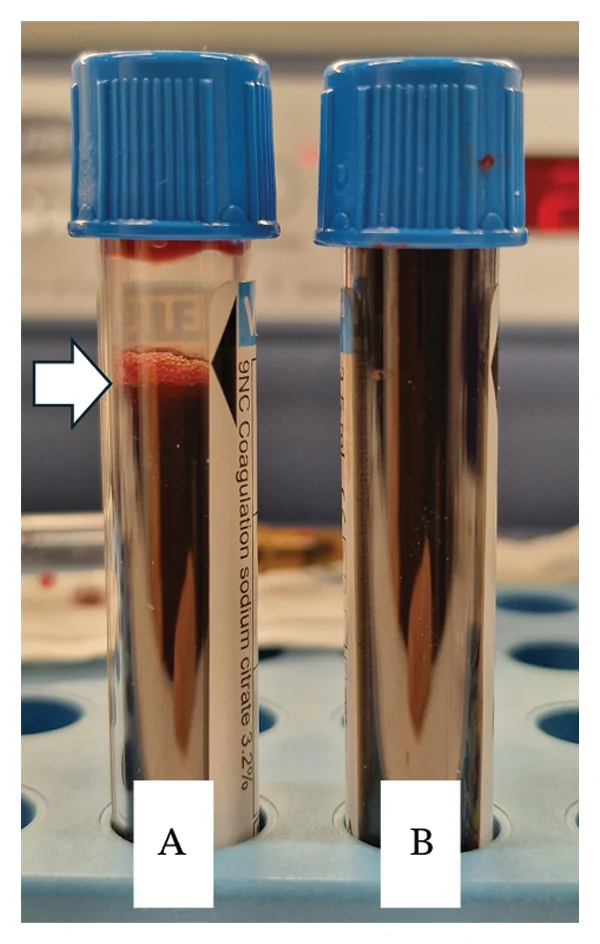
Standard tubes and fully filled tubes. Samples were drawn into vacuum tubes using a standard technique, leaving a blood–air interface in the tube (white arrow in tube (A)) or the tubes were completely filled with blood following vacuum draw (tube (B)).

Five citrate and subsequently five EDTA tubes were filled following the standard venipuncture procedure, leaving a blood–air interface within the tubes (Figure 1(A)). In contrast, another set of five citrate and subsequently five EDTA tubes were filled completely with blood, eliminating the blood–air interface (Figure 1(B)) using the following procedure: Blood was drawn into eight citrate and subsequently eight EDTA tubes using vacuum according to standard procedure. After the tubes had filled to the indicated fill line, the lids were removed, and five tubes were filled completely by gentle pipetting blood from the additional three citrate or EDTA tubes, respectively, thereby retaining the anticoagulation‐to‐blood ratio. All visible microbubbles were aspirated from the blood, and the tubes were recapped with Henke‐Ject 26G syringes (Henke Sass Wolf, Tuttlingen, Germany) inserted through the tube cap to allow drainage of excess blood and air. The syringes were removed at the start of the incubation.

All tubes were incubated at room temperature (approximately 21°C–25°C) on a Stuart Microtiter plate shaker SSM5 (Cole‐Parmer, Vernon Hills, IL) at 250 × *g*, to simulate sample transportation.

Immediately after blood drawing and after 2, 4, 6, 24, and 48 h of incubation, a set of citrate and EDTA tubes with and without air was removed from the plate shaker. The EDTA tubes were centrifuged at 3000 × *g* for 20 min. at 4°C, and plasma was transferred to 1‐mL polypropylene Matrix ScrewTop Tubes (Thermo Scientific, Hudson, NH) and stored at −80°C. Prior to the ROTEM analysis, the citrate tubes were carefully inverted five times to resuspend the sedimented blood cells.

### 2.2. ROTEM Analysis

After tilting, the citrate tubes were incubated for 5 minutes at 37°C on a heater block. Coagulation kinetics were analyzed on a ROTEM Delta instrument (Werfen, München, Germany) using EXTEM and INTEM reagents as per the manufacturer’s instructions. After pipetting blood for the ROTEM analysis, the citrate tubes were centrifuged at 2000 × *g* for 15 min. at room temperature. Plasma was transferred to Matrix tubes and stored at −80°C.

### 2.3. Plasma Coagulation Analysis

Citrate plasma was thawed on ice water and analyzed for APTT and INR on the Siemens Sysmex CS‐5100 (Siemens Healthineers, Erlangen, Germany) and for prothrombin fragment 1 + 2 (PTF1 + 2) using the Enzygnost F1 + 2 kit (Siemens Healthcare, Marburg, Germany) as per the manufacturer’s instructions. The absorbance (optical density) of the ELISA plates was read using a Tecan Infinite M200 plate scanner (Tecan Group, Männedorf, Switzerland) and the Magellan 7.1 SP1 software (Tecan Group) with settings as per the manufacturer’s instructions.

### 2.4. Platelet Markers

EDTA plasma from six donors (three male and three female) from incubations in EDTA tubes was thawed on ice water and analyzed for β‐thromboglobulin (BTG) using the Human CXCL7/NAP‐2 DuoSet ELISA kit (R&D Systems, Abingdon, UK) and for sP‐selectin using the Invitrogen human sP‐selectin ELISA kit (Thermo Fisher Scientific). Both analyses were performed as per the manufacturer’s instructions, and the absorbance of the ELISA plates was read using the Tecan Infinite M200 plate scanner and the Magellan 7.1 SP1 software.

### 2.5. Blood Gas Analysis

From four donors (two male and two female), 1 mL of blood from citrate and EDTA samples—with and without air—was transferred into SafePICO heparinized blood gas syringes (Radiometer, Brønshøj, Denmark) at baseline and after 2, 4, 6 (citrate only), 24, and 48 h of incubation. Samples were analyzed for pH, partial pressure of CO_2_ (pCO_2_), partial pressure of O_2_ (pO_2_), bicarbonate, hemoglobin, sodium, potassium, calcium, glucose, and lactate using a Radiometer ABL800 Flex blood gas analyzer, following the manufacturer’s instructions.

### 2.6. Hemolysis Analysis

To assess for hemolysis in the EDTA plasma, plasma from six donors (three male and three female) from incubations in EDTA tubes was analyzed for plasma free hemoglobin by spectrophotometry using the HemoCue Plasma/Low Hb system (HemoCue AB, Ängelholm, Sweden), measuring absorbance at 570 and 880 nm, and the NanoDrop microvolume spectrophotometer (Thermo Fisher Scientific, Waltham, MA), measuring absorbance at 414 and 600 nm. Additionally, cell lysis was assessed by measuring lactate dehydrogenase enzyme activity in the plasma samples on the Atellica Clinical Chemistry Analyzer (Siemens Healthineers).

### 2.7. Data Handling and Statistical Analysis

The power of the study was not calculated, as this was an exploratory study designed to investigate preanalytical stability under different conditions. The primary aim was to generate preliminary data and to identify trends that may inform future, larger‐scale studies.

We organized data using Excel for Mac 16.69.1 (Microsoft Corporation, Richmond, WA) and conducted statistical analyses and graphing using Prism for Mac 10.0.3 (GraphPad Software, La Jolla, CA). One ROTEM EXTEM CFT result from a four‐hour full tube incubation was missing due to technical problems with the instrument. This value was imputed using the average data for similar incubations.

To investigate the effects of incubation time and standard versus full tubes on various parameters, we employed repeated measures of two‐way ANOVA with uncorrected Fisher’s LSD multiple comparison tests for APTT, INR, BTG, sP‐selectin, and ROTEM. We calculated Pearson correlations to explore relationships between LDH, BTG, and sP‐selectin, as well as between LDH, HemoCue, and NanoDrop measurements. A significance level of *p* < 0.05 was applied, and data were presented as medians with interquartile range (IQR) for ROTEM, BTG, sP‐selectin, and hemolysis due to non‐Gaussian distribution and as means with 95% confidence interval (CI) for APTT, INR, and PTF1 + 2. When possible, we also compared the bias of the analytes with the recommended analytical quality specifications according to the new guidelines from the EFLM biological variation database [4, 5]. Focusing on maximum acceptable bias according to Model 2, we calculated the maximum acceptable bias as 0.375 times the total biological variation. The calculated maximum acceptable bias of selected analytes is included in Supporting Table 1.

## 3. Results

### 3.1. ROTEM

In blood drawn and stored in standard Vacuette citrate tubes, without removing ambient air from the tube (white circles in Figure 2), the ROTEM readouts EXTEM CT (Figure 2(a)) and EXTEM CFT (Figure 2(b)) remained within eleven percent of baseline values throughout the 48 h of incubation. EXTEM ɑ‐angle (Figure 2(c)), EXTEM MCF (Figure 2(d)), INTEM ɑ‐angle (Figure 2(g)), and INTEM MCF (Figure 2(h)) remained within five percent of baseline values throughout the 48 h of incubation (Table 1). The INTEM CT (Figure 2(e)) and INTEM CFT (Figure 2(f)) remained within ten percent of baseline values throughout the six hours of incubation, and within 17 percent throughout the 48 h of incubation. EXTEM CFT exceeded the maximum acceptable bias (as described in Supporting Table 1) after 2 hours, EXTEM MCF after 24 h, and EXTEM CT after 48 h of incubation. All other ROTEM readouts remained within the maximum acceptable bias throughout the 48 h of incubation. In contrast, for blood stored in full tubes, without any air in the tube (gray circles in Figure 2), both EXTEM CFT (Figure 2(b)) and INTEM CFT (Figure 2(f)) were reduced ≧ 10% after 4 hours (*p* = 0.005 and *p* = 0.02) (Table 1). In blood stored in full tubes, the EXTEM CFT and MCF exceeded the maximum acceptable bias after 4 hours, the EXTEM CT after 6 hours, and the INTEM CFT after 48 h of incubation (Table 1).

FIGURE 2Coagulation kinetics analyzed by ROTEM. Coagulation was measured using ROTEM in blood (*n* = 10, five male, five female) incubated in citrated Vacuette tubes filled using standard technique (white circles) and in tubes filled using standard technique and subsequently topped up with citrated blood and capped, ensuring no air was present inside the tube (gray circles). Tubes were incubated upright for 48 h at room temperature on a shaker running at 250 rpm. After 0, 2, 4, 6, 24, and 48 h, a set of samples were analyzed using the EXTEM (containing tissue factor as coagulation activator, (a–d)) and the INTEM (containing phospholipid and ellagic acid as coagulation activators, (e–h)) chemistry. The horizontal lines are medians, and error bars span the 25th to the 75th percentile. ROTEM rotational thromboelastometry, CT clotting time, CFT clot formation time, MCF maximum clot formation. Changes from baseline in standard tubes and differences between standard tubes and full tubes were compared using RM‐ANOVA with uncorrected Fisher’s LSD test. Only significant (*p* < 0.05) differences are shown on the graphs.

**Figure:**
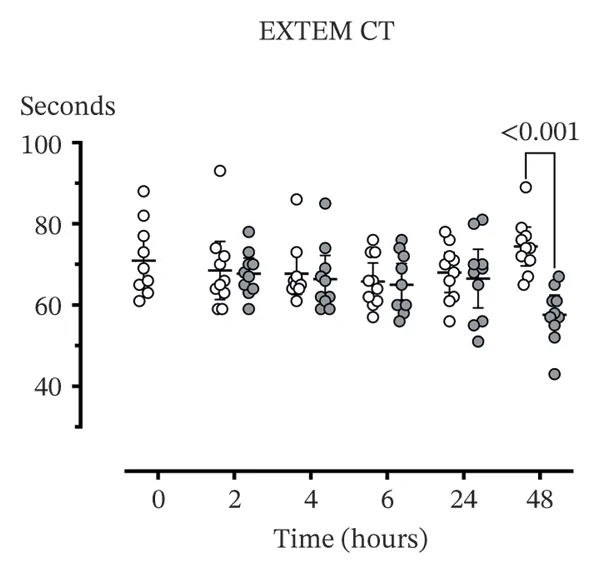
(a)

**Figure:**
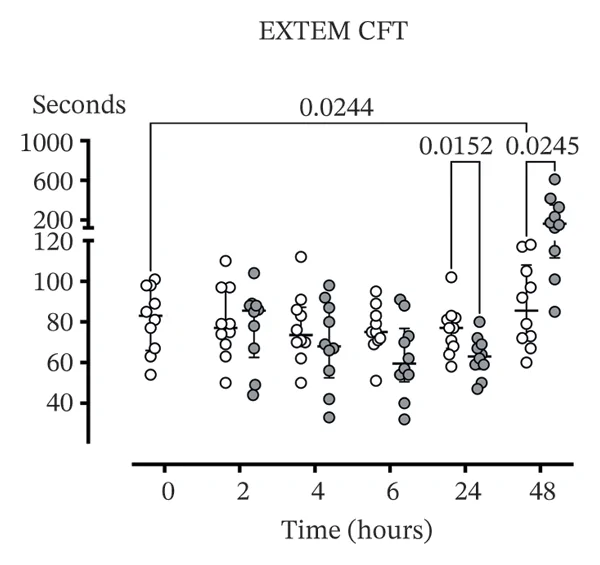
(b)

**Figure:**
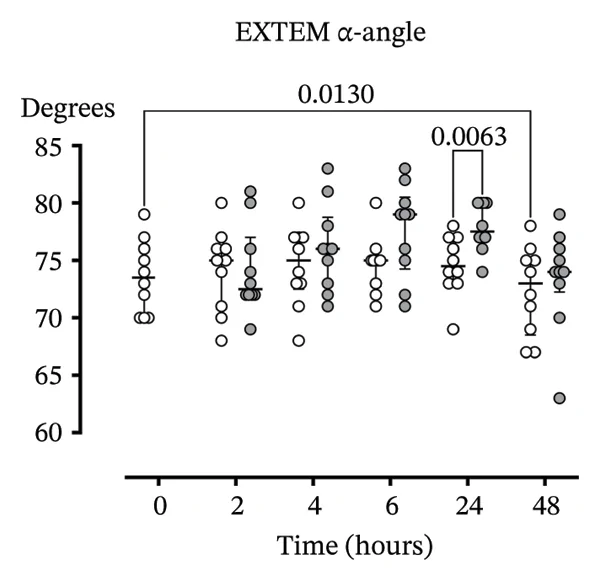
(c)

**Figure:**
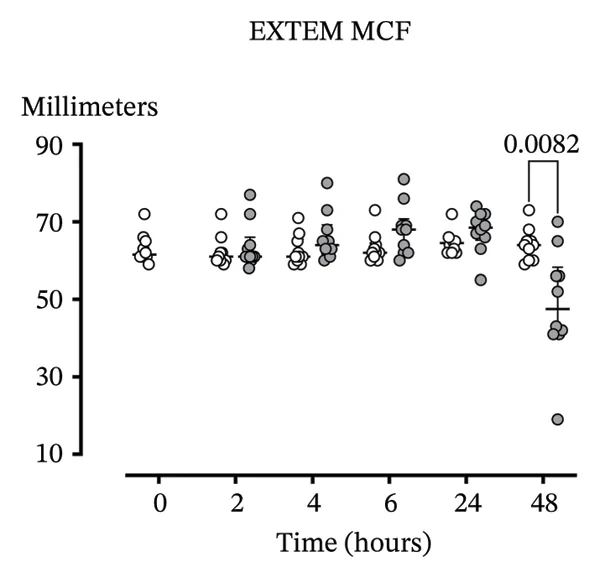
(d)

**Figure:**
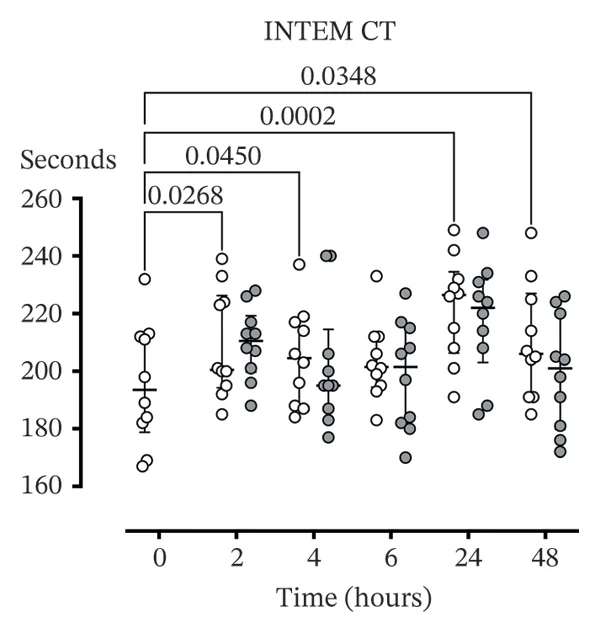
(e)

**Figure:**
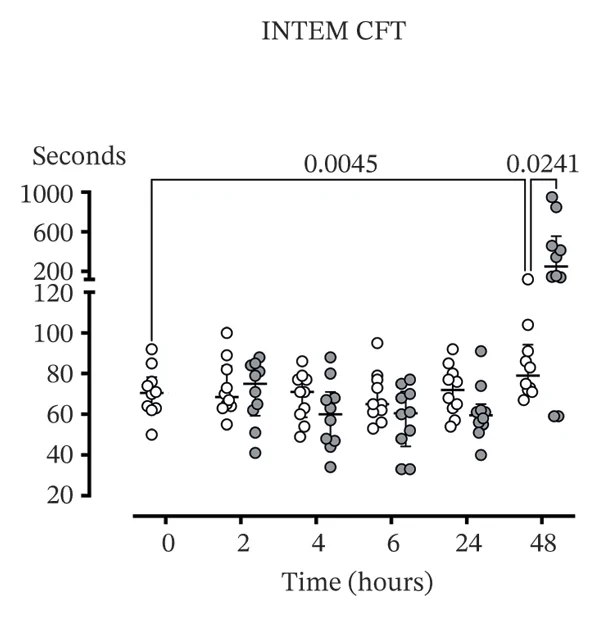
(f)

**Figure:**
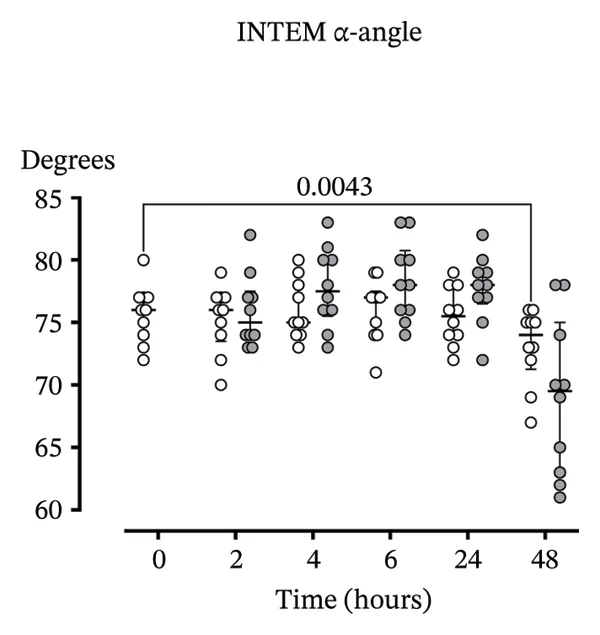
(g)

**Figure:**
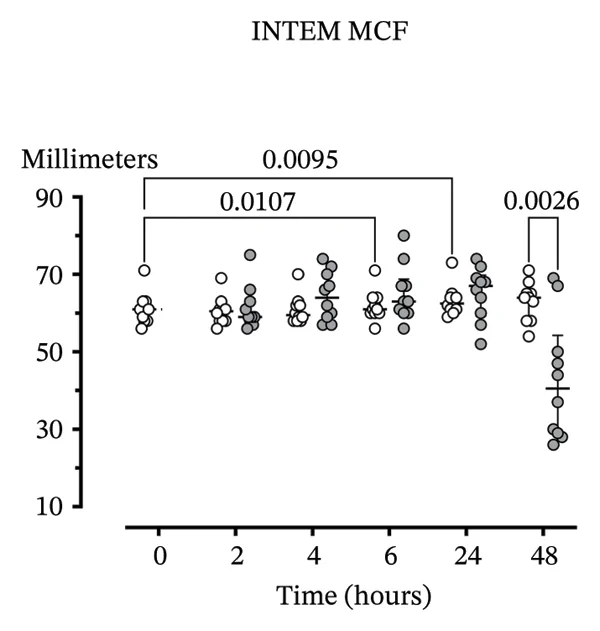
(h)

**TABLE 1:** Effect of preanalytical storage time and air on ROTEM readouts.

Sample storage time (hours)	2	4	6	24	48
*EXTEM, standard tubes*					
CT (s)	−3.0	−3.0	−3.7	2.2	9.6^↑^
CFT (s)	−7.2^**↑**^	−11^↑^	−9.6^↑^	−7.2^↑^	**3.0**
ɑ‐Angle (degrees)	2.0	2.0	2.0	1.4	−**0.7**
MCF (millimeters)	−0.8	−0.8	0.8	4.9^↑^	4.1^**↑**^

*INTEM, standard tubes*					
CT (s)	**3.6**	**5.7**	4.1	**17**	**6.5**
CFT (s)	−2.8	0.7	−7.8	2.1	**12**
ɑ‐Angle (degrees)	0.0	−1.3	1.3	−0.7	−**2.6**
MCF (millimeters)	−0.8	−2.5	**0**	**2.5**	4.9

*EXTEM, full tubes*					
CT (s)	0	−**5.2**	−**7.4** ^**↑**^	**1.5** ^**↑**^	−**15** ^∗↑^
CFT (s)	−3.0	−**18** ^**↑**^	−**28** ^**↑**^	−**24** ^**∗↑**^	**95** ^**∗↑**^
ɑ‐Angle (degrees)	−1.4	**3.4**	**7.5**	**5.4** ^∗^	0.7
MCF (millimeters)	0.8	**4.1** ^**↑**^	**11** ^↑^	11^↑^	−**23** ^**∗↑**^

*INTEM, full tubes*					
CT (s)	**8.8**	0.8	4.1	**15**	3.9
CFT (s)	6.4	−**15**	−**14**	−16	**254** ^**∗↑**^
ɑ‐Angle (degrees)	0.0	−**1.3**	**2.0**	2.6	−**8.6**
MCF (millimeters)	−3.3	**4.9**	**3.3**	9.8	−**34** ^∗^

*Note:* Change from median baseline values (%). Significant changes (*p* < 0.05) from baseline values are shown in bold. Significant differences (*p* < 0.05) between full and standard tubes are marked with^∗^. Changes exceeding the maximum acceptable bias (described in Supporting Table 1) are marked with^↑^. Maximum acceptable bias is undefined for INTEM MCF, INTEM ɑ‐angle, and EXTEM ɑ‐angle. ROTEM, rotational thromboelastometry; s, seconds.

Abbreviations: CFT, clot formation time; CT, clotting time; MCF, maximum clot formation.

### 3.2. Coagulation and Fibrinolysis

In citrate plasma, baseline APTT was 25 (24–27) s (Figure 3(a)). APTT remained at baseline values during 6 hours of incubation and within nine percent of baseline during 24 h of incubation (Table 2). After 48 h of incubation, APTT had increased 16% above baseline values to 29 (27–31) s (*p* < 0.0001). The maximum acceptable bias for APTT of 1.9% (Supporting Table 1) was exceeded after 24 h of incubation (Table 2). No significant difference in APTT was observed between samples incubated in standard tubes and in full tubes without air (Figure 3(a) and Table 2).

FIGURE 3Coagulation and fibrinolytic markers. The coagulation analysis activated partial thromboplastin time (APTT), prothrombin international normalized ratio (INR), and prothrombin fragment 1 + 2 (PTF1 + 2) were measured in plasma from blood (*n* = 10, five male, five female) incubated in citrated Vacuette tubes filled using standard technique (white circles) and in tubes filled using standard technique and subsequently topped up with citrated blood and capped, ensuring no air was present inside the tube (gray circles). Tubes were incubated upright for 48 h at room temperature on a shaker running at 250 rpm. After 0, 2, 4, 6, 24, and 48 h, a set of samples were analyzed for APTT (a) and INR (b) in the routine laboratory and for PTF1 + 2 using ELISA (c). Horizontal lines are means, and error bars span the 95% CI. Changes from baseline in standard tubes and differences between standard tubes and full tubes were compared using RM‐ANOVA with uncorrected Fisher’s LSD test. Only significant (*p* < 0.05) differences are shown on graphs.

**Figure:**
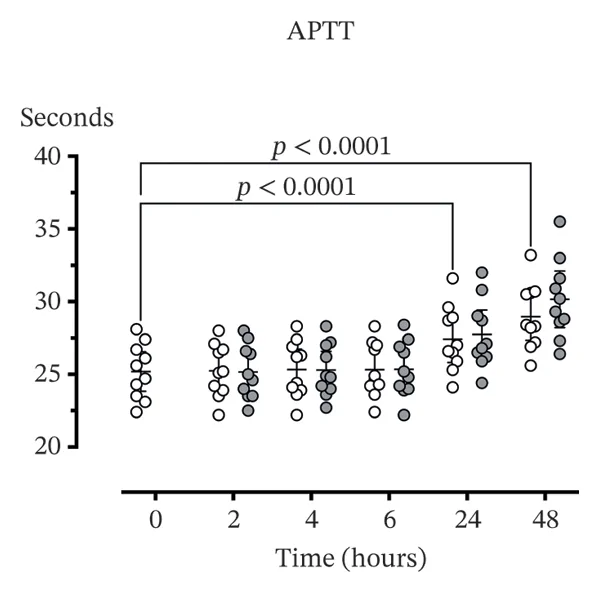
(a)

**Figure:**
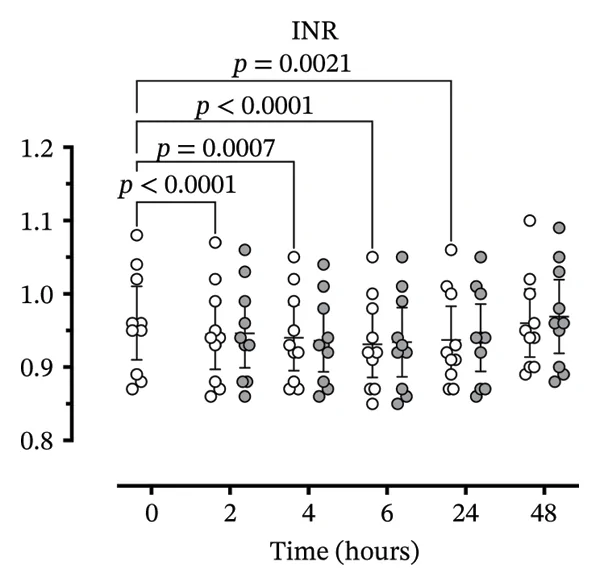
(b)

**Figure:**
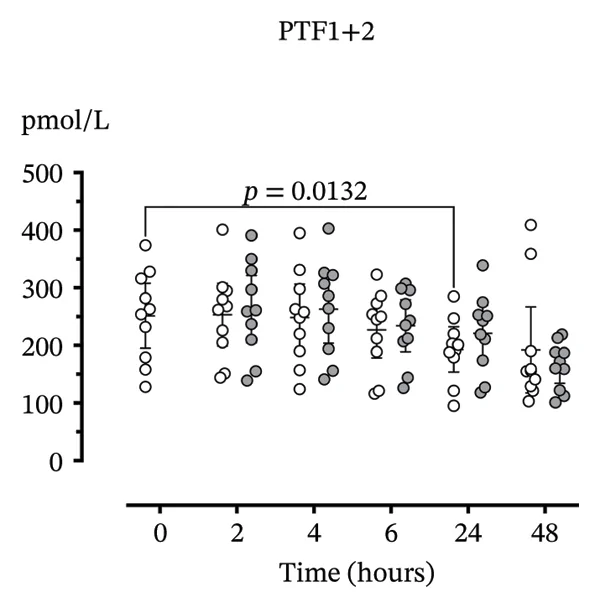
(c)

**TABLE 2:** Effect of preanalytical storage time and air on coagulation and platelets^a^.

Sample storage time (hours)	2	4	6	24	48
*Standard tubes*					
APTT	0.2	0.6	0.5	**8.9** ^**↑**^	**15** ^**↑**^
INR	−**1.6** ^**↑**^	−**2.1** ^**↑**^	−**3.0** ^**↑**^	−**2.4** ^**↑**^	0
PTF1 + 2	0.8	−1.2	−10	−**23**	−24
BTG	395	431	498	1183	**2055**
sP‐selectin	8.9	24^**↑**^	20^**↑**^	**32** ^**↑**^	**53** ^**↑**^

*Full tubes*					
APTT	−0.1	0.4	0.6	**10** ^**↑**^	**20** ^**↑**^
INR	−**1.5** ^**↑**^	−**2.5** ^**↑**^	−**2.7** ^**↑**^	−**2.1** ^**↑**^	**0.9**
PTF1 + 2	4.6	4.6	−6.9	−12	−**35**
BTG	555	671	734	**1410**	**3370**
sP‐selectin	**15** ^**↑**^	**21** ^**↑**^	**23** ^**↑**^	**29** ^**↑**^	**94** ^**↑**^

*Note:* Change from baseline values (%). BTG and sP‐selectin are median changes in EDTA blood from six donors. Significant changes (*p* < 0.05) from baseline values are shown in bold. Significant differences (*p* < 0.05) between full and standard tubes are marked with^∗^. Changes exceeding maximum acceptable bias (Supporting Table 1) are marked with^↑^. Maximum acceptable bias is undefined for BTG and PTF1 + 2. BTG, β‐thromboglobulin; INR, prothrombin time international normalized ratio; PTF1 + 2, prothrombin fragment 1 + 2.

Abbreviations: APTT, activated partial thromboplastin time.

^a^APTT, INR, and PTF1 + 2 are average changes in citrate blood from ten donors.

The baseline INR was 1.0 (0.9–1) (Figure 3(b)). The INR remained within 3% of baseline throughout the 48 h of incubation (Figure 3(b) and Table 2), but exceeded the maximum acceptable bias of 1.3% already after 2 hours (Table 2). No significant difference in INR was observed between samples incubated in standard tubes and in full tubes (Figure 3(b) and Table 2).

Baseline PTF1 + 2 was 251 (195–308) pmol/L (Figure 3(c)). PTF1 + 2 remained within ten percent of baseline during the first 6 hours of incubation (Figure 3(c) and Table 2). After 24 and 48 h of incubation, PTF1 + 2 was significantly reduced (*p* = 0.01) with 23% from baseline (Figure 3(c) and Table 2). The maximum acceptable bias is undefined for PTF1 + 2 due to lack of biological variation data. No significant difference in PTF1 + 2 between blood samples incubated in standard tubes and in full tubes was noted throughout the 48 h of incubation (Table 2).

### 3.3. Platelet Activation

In blood drawn on standard Vacuette EDTA tubes, the baseline BTG was 63 (58–83) ng/mL (Supporting Figure 1(A)). After two hours of incubation, BTG increased 395 percent (*p* = 0.1) (Supporting Figure 1(A) and Table 2). BTG remained at this level throughout the six hours of incubation and increased further to 1183% of baseline (*p* = 0.06), after 24 h of incubation, and to 2055 percent of baseline after 48 h of incubation (*p* = 0.01). The maximum acceptable bias is undefined for BTG since the biological variation is not known.

Baseline sP‐selectin was 40 (37–55) ng/mL (Supporting Figure 1(B)). In standard tubes, sP‐selectin remained within 10% of baseline levels during the first 2 hours of incubation. After 24 h, sP‐selectin increased 32% to 53 (43–84) ng/mL (*p* = 0.03). sP‐selectin exceeded the maximum acceptable bias after 4 hours of incubation (Table 2). No significant differences in BTG or sP‐selectin between standard and full tubes were observed throughout the 48 h of incubation (Table 2). BTG and sP‐selectin levels correlated moderately (*r* = 0.34, *p* = 0.005).

### 3.4. Blood Gases and Metabolites

#### 3.4.1. Citrate Blood

In blood incubated in standard Vacuette citrate tubes, baseline pH was 7.21 (95% CI 6.94–7.47; Supporting Figure 3A). pH dropped throughout the incubation (Table 3). After 6 hours of incubation, pH had dropped to 7.06 (95% CI 7.0–7.1; *p* = 0.03). Baseline pO_2_ was 3.9 (95% CI 2.9–4.9) kPa (Supporting Figure 3(B)). pO_2_ remained within 20% of baseline levels throughout the 48 h of incubation (Table 3). Baseline pCO_2_ was 7.2 (95% CI 2.2–12) kPa (Supporting Figure 3(C)). pCO_2_ increased throughout the incubation (Table 3). After 2 hours of incubation, pCO_2_ had increased 27% above baseline. Baseline bicarbonate was 19 (95% CI 13–24) mmol/L (Supporting Figure 3(D)). Bicarbonate remained within ten percent of baseline levels throughout the 48 h of incubation (Table 3). Baseline hemoglobin was 13 (95% CI 11–15) g/dL (Supporting Figure 3(E)). Hemoglobin remained within ten percent of baseline throughout the 48 h of incubation (Table 3). Baseline potassium was 3.3 (95% CI 3.1–3.4) mmol/L (Supporting Figure 3(F)). Potassium remained within five percent of baseline values during 24 h of incubation and increased to 6.6 (5.9–7.3) mmol/L after 48 h of incubation (Table 3). Baseline glucose was 4.8 (95% CI 3.4–6.1) mmol/L (Supporting Figure 3(G)). Glucose decreased throughout the incubation (Table 3). After 2 hours, glucose had dropped 14% from baseline values. Baseline lactate was 0.8 (95% CI 0.7–1.0) mmol/L (Supporting Figure 3(H)). Lactate increased throughout the incubation (Table 3). After 2 hours, lactate had increased by 136%. No significant differences between blood samples incubated in standard tubes and in full tubes were noted for any of these readouts (Table 3 and Supporting Figures 3(A)–3(H)).

**TABLE 3:** Effect of preanalytical storage time and air in tubes on blood gas and metabolites.

Sample storage time (hours)	Standard tubes			Full tubes		
	6	24	48	6	24	48
*Vacuette citrate tubes*						
pH	−2^↑^	−**3.7** ^**↑**^	−**4.7** ^**↑**^	−1.5^↑^	−**4.2** ^**↑**^	−**5.3** ^**↑**^
pO_2_	18^↑^	7.5^↑^	−**9** ^**↑**^	48^↑^	**15** ^**↑**^	−**8.3** ^**↑**^
pCO_2_	39^↑^	63^↑^	92^↑^	28^↑^	71^↑^	93^↑^
Bicarbonate	8.7^↑^	−6.1^↑^	−5.4^↑^	5.3^↑^	−7.3^↑^	−14^↑^
Hemoglobin	9.4^↑^	0.4	0.6	−23^↑^	−22^↑^	−**49.6** ^**∗↑**^
Potassium	2.3^↑^	**23** ^**↑**^	**101** ^**↑**^	0.8^↑^	19^↑^	**60** ^**∗↑**^
Glucose	−**28** ^**↑**^	−**72** ^**↑**^	−**86** ^**↑**^	−**21** ^**↑**^	−**81** ^**↑**^	−**97** ^**↑**^
Lactate	276^↑^	**785** ^**↑**^	**1021** ^**↑**^	**256** ^**∗↑**^	**930** ^**↑**^	**1163** ^**↑**^

*Vacuette EDTA tubes*						
pH	−**1.9** ^**↑**^	−**3.8** ^**↑**^	−**4.9** ^**↑**^	−**2** ^**↑**^	−**4.8** ^**↑**^	−**5.9** ^**↑**^
pO_2_	−11^↑^	−15^↑^	−44^↑^	55^↑^	−26^↑^	−44^↑^
pCO_2_	**48** ^**↑**^	**81** ^**↑**^	**122** ^**↑**^	**50** ^**↑**^	**111** ^**∗↑**^	**144** ^**↑**^
Bicarbonate	**9.3** ^**↑**^	−2.8^↑^	−1.4	7.9^↑^	−3.5^↑^	−8.7^↑^
Hemoglobin	12^↑^	−4^↑^	−**1.2**	−15^↑^	−14^↑^	−28^∗↑^
Potassium	−**6.4** ^**↑**^	−0.1	5^↑^	−3.2^↑^	−1.9	3^∗↑^
Glucose	−**23** ^**↑**^	−66^↑^	−**84** ^**↑**^	−**31** ^**↑**^	−**91** ^**↑**^	−**100** ^**↑**^
Lactate	**280** ^**↑**^	**741** ^**↑**^	**836** ^**↑**^	**328** ^**∗↑**^	**902** ^**↑**^	**1056** ^**↑**^

*Note:* Change from baseline values (%). Changes from baseline and differences between standard and full tubes were compared using a mixed‐effects model. Significant changes from baseline values (*p* < 0.05) are shown in bold. Significant differences (*p* < 0.05) between standard and full tubes are marked with ^∗^. Changes exceeding maximum acceptable bias (see Supporting Table 1) are marked with^↑^. Bold values represent significant changes from baseline values ().

#### 3.4.2. EDTA Blood

In blood incubated in standard Vacuette EDTA tubes, baseline pH was 7.16 (95% CI 7.07–7.24; Supporting Figure 4(A)). pH dropped throughout the incubation (Table 3). After 6 hours of incubation, pH had dropped to 7.02 (95% CI 6.96–7.03; *p* = 0.006). Baseline pO_2_ was 6.5 (95% CI 2–11) kPa (Supporting Figure 4(B)). pO_2_ remained within 15% of baseline levels throughout the 24 h of incubation (Table 3). After 48 h, pO_2_ had dropped by 44%. Baseline pCO_2_ was 8.5 (95% CI 5.1–11) kPa (Supporting Figure 4(C)). pCO_2_ increased throughout the incubation (Table 3). After 2 hours of incubation, pCO_2_ had increased 48% above baseline. Baseline bicarbonate was 20 (95% CI 16–24) mmol/L (Supporting Figure 4(D)). Bicarbonate remained within ten percent of baseline levels throughout the 48 h of incubation (Table 3). Baseline hemoglobin was 14 (95% CI 12–17) g/dL (Supporting Figure 4(E)). Hemoglobin remained within 12% percent of baseline throughout the 48 h of incubation. Baseline potassium was 24 (95% CI 21–24) mmol/L (Supporting Figure 4(F)). Potassium remained within seven percent of baseline values throughout the 48 h of incubation (Table 3). Baseline glucose was 5.4 (95% CI 3.9–6.9) mmol/L (Supporting Figure 4(G)). Glucose decreased throughout the incubation (Table 3). After 2 hours, glucose had dropped 23% from baseline values. Baseline lactate was 1.0 (95% CI 0.6–1.3) mmol/L (Supporting Figure 4(H)). Lactate increased throughout the incubation (Table 3). After 2 hours, lactate had increased by 280%. No significant differences between blood samples incubated in standard tubes and in full tubes were noted for any readout (Table 3 and Supporting Figure 4(A)–4(H)).

Apart from potassium, the results for blood gases and metabolites in citrated blood (Supporting Figure 3) and EDTA blood (Supporting Figure 4) were nearly indistinguishable. Of note, nearly all blood gas and metabolite analysis in both citrated and EDTA‐treated blood with and without air exceeded the maximum acceptable bias already after 2 hours of incubation (Table 3).

### 3.5. Hemolysis and Cell Death

Plasma free hemoglobin level measured by HemoCue Low Hb (Supporting Figure 2A) and absorbance by NanoDrop spectrophotometer at 414 and 600 nm (Supporting Figures 2B and 2C) remained at baseline levels throughout the 48 h of incubations (all *p* > 0.05). LDH levels, indicative of cell death beyond just hemolysis, remained stable during the initial 6 hours of incubation (Supporting Figure 2(D)). However, they exhibited a significant rise after 24 h, which increased further after 48 h of incubation (*p* < 0.02). No differences in plasma free hemoglobin, absorbance, or LDH between blood samples incubated in standard tubes and in tubes filled with blood were observed throughout the incubation period (all *p* > 0.05) (Supporting Figures 2(A)–2(D)).

Plasma free hemoglobin, as measured by HemoCue, exhibited a strong positive correlation with absorbance measured by NanoDrop at 414 nm (*r* = 0.82, *p* < 0.001) and at 600 nm (*r* = 0.67, *p* < 0.001). Additionally, NanoDrop absorbance at 414 and 600 nm showed a strong positive correlation (*r* = 0.88, *p* < 0.001). In contrast, no significant correlations were found between LDH levels and HemoCue or NanoDrop measurements, consistent with the pronounced increase in LDH levels observed after 24 and 48 h. LDH levels correlated moderately with BTG levels (*r* = 0.44, *p* = 0.0003) and weakly with sP‐selectin (*r* = 0.33, *p* = 0.006).

## 4. Discussion

In the present study, we found that the ROTEM analyses demonstrated a longer stability period than the previously estimated two‐hour timeframe indicated in the literature [14]. Generally, except for INTEM CT, all measurements remained stable for up to 24 h of storage and stayed within 16% of baseline measurements after 48 h. However, EXTEM CFT exceeded the maximum recommended bias already after 2 hours (nonsignificant finding). While the manufacturer gives no specific recommendations regarding the preanalytical sample storage time [15], previous studies have found that ROTEM coagulation kinetics may reliably be analyzed in blood stored for up to 6 hours [16]. ROTEM analyses are generally recommended immediately or within 2 hours after blood collection [14], and samples should not be sent through a pneumatic tube transportation system to avoid platelet activation [12], putting high demands on laboratory logistics. However, we found only slight changes in the ROTEM parameters during 6 hours of storage and shaking, indicating that citrate blood samples can be stored and transported before analysis, potentially expanding the practical utility of this analysis. The storage stability of citrate samples for ROTEM analysis is relevant as samples are often transported to a central laboratory in many hospitals. Additionally, ROTEM instruments have limited sample capacity, meaning that the analysis of some samples may be delayed even when the instrument is placed in an emergency department. This stability may be particularly advantageous in settings with long transport times, shared laboratory resources, or research contexts, allowing for more flexible and efficient sample handling.

The within‐subject biological variation (CVi) and analytical imprecision (CVa) can be used to calculate reference change values (RCVs) to assess the probability that a difference between two consecutive measurements in one patient can be explained by analytical and within‐subject biological variation. In this study, we calculated the maximum acceptable bias from the total biological variation for several analytes and compared it with the significant changes in values over time. As an alternative to the RCV, the maximum acceptable bias, often calculated as 0.375 times the total biological variation of the analyte, is often used as a quality goal, and valid conclusions based on a laboratory result must not exceed the maximum acceptable bias [17]. In our study, we found several analytes, INR and some ROTEM readouts, although nonsignificantly, exceeded the maximum acceptable bias already after 2 hours.

APTT and PTF1 + 2 were stable over the first 6 hours, and INR was stable for 48 h. The observed stability of APTT for up to 6 hours at room temperature with sample shaking exceeded expectations. However, this may be due to the limited number (*n* = 10) of samples studied. Norwegian guidelines and recommendations from the Clinical Laboratory Standards Institute H21‐A5 guideline suggest analyzing APTT within two to four hours to prevent potential errors related to the Factor VIII and Factor V depletion [18, 19]. Although the INR exceeded the referenced maximum acceptable bias of 1.3% within 2 hours, these changes are clinically irrelevant. The INR remained stable within 3% of baseline throughout the 48‐hour period, consistent with findings from prior studies and national Norwegian recommendations [20]. The degree of bias of INR was clinically insignificant, but the degree of shaking of these samples in our in vitro study may be lower than experienced by clinical samples, for example, in pneumatic tube transportation. We speculate that the slight changes in the INR and APTT after 24 and 48 h of storage may be partly due to the slight shaking experienced by the samples and partly due to contact activation of coagulation in the sample tube.

Blood gases and metabolite levels exhibited immediate changes, with either increases or decreases, within the first 2 to 6 hours after blood sampling, in accordance with established knowledge. Notably, a nearly identical pattern was observed for both citrated and EDTA blood samples. However, only heparinized blood is used for routine analysis of blood gases. Interestingly, pH and glucose levels decreased while lactate levels increased during incubation, regardless of citrate or EDTA use, suggesting that neither anticoagulant significantly attenuated blood metabolism. Consequently, blood gases and metabolites should be analyzed promptly using heparinized blood since both EDTA and citrate affect calcium measurements.

Concerning platelet activation, BTG, stored within alpha‐granules and released upon platelet activation, showed a substantial 395% increase after 2 hours of incubation. However, there was only minimal further increase in BTG levels during the subsequent 6 hours, indicating that platelet activation is most likely triggered by the initial sample handling and contact activation within the sample tube rather than the subsequent shaking and duration of storage, including the drop in pH. Similarly, sP‐selectin, a protein released during platelet activation, either by proteolytic cleavage of the transmembrane P‐selectin protein [21] or, to some extent, by the release within alpha‐granules [22], showed a minor 9% increase during the first 2 hours of incubation and then remained relatively stable for the next 6 hours before increasing further. The disparity in the extent of increase between BTG and sP‐selectin may be attributed to unchanged proteolytic cleavage of the P‐selectin within platelet membranes.

When EDTA blood samples were stored for up to 48 h, there was minimal hemolysis as assessed by both HemoCue and NanoDrop, which correlated well. However, LDH levels showed a significant increase after 24 h and continued to rise at 48 h, indicating cell death. The correlation between LDH, BTG, and sP‐selectin levels suggests that LDH at least partially reflects platelet lysis and the subsequent protein release. Notably, this platelet lysis seems to be exacerbated when air is removed from the tubes. Hemolysis may affect several laboratory analyses and should be avoided and managed according to the hemolysis (H)‐index results on routine instruments [23]. In EDTA blood, hemolysis measured by absorbance with HemoCue and NanoDrop was minimal throughout 48 h, suggesting good red cell stability in the samples. Results from HemoCue strongly correlated with NanoDrop at 414 nm and moderately at 600 nm, highlighting the usefulness of both methods for assessing hemolysis in samples. In contrast, LDH did not correlate with NanoDrop or HemoCue, suggesting that LDH is not a specific marker of hemolysis in vitro. This indicates that the observed LDH increase likely originated from cells other than hemolyzed red cells, possibly leucocytes. However, LDH levels showed a significant increase after 24 h and continued to rise at 48 h, indicating the occurrence of cell death. The correlation between LDH, BTG, and sP‐selectin levels is consistent with a general activation and possible cell death of platelets occurring after 24 h. Thus, to mitigate the effects of cell lysis during sample storage and transportation, it is imperative to remove cells by centrifuging the samples within 6 hours.

The removal of air from the sample tubes did not significantly extend preanalytical storage time for any of the tested readouts, contrary to our hypothesis that mitigating air would enhance sample stability. This may be due to blood activation during sample acquisition and preparation in both standard and filled tubes and pipetting and recapping in filled tubes. We employed a standard clinical laboratory technique, drawing blood with a PVC tubing blood collection set, which is known to activate cascade systems [24, 25]. Additionally, vacuum‐based sampling methods like the Vacutainer system can activate the platelets through shear stress exposure during blood sampling [26], and improper tube filling is linked to increased hemolysis [27]. We observed bubbles and foam in several of the Vacuette tubes, likely due to a strong vacuum. Thorough, yet careful, pipetting was required to remove bubbles from blood incubated in full tubes, potentially further activating the platelets and cascades.

### 4.1. Limitations

Our study faced several limitations, primarily attributed to the relatively small dataset. BTG, sP‐selectin, and hemolysis data were gathered from only six donors. This limited sample size amplified the influence of outliers on our results and statistical analyses. Furthermore, the limited sample size led to some trends lacking statistical significance, which calls for caution when interpreting the findings and raises the possibility of Type II errors. Furthermore, our findings may not be generalizable, as this was a single‐center study, and sample handling and analysis can vary across different environments and hospital settings. Nevertheless, it is worth noting that the biological effect of these minor differences might be negligible. Samples were only analyzed once, preventing any assessment of potential intra‐assay variability. Additionally, hemolysis was only assessed in EDTA samples, making it impossible to draw comparisons with citrate samples.

## 5. Conclusion

In this single‐center explorative study, most ROTEM analyses remained reliable after 6 hours and INR even after 48 h of storage. APTT, PTF1 + 2, BTG, and sP‐selectin may be analyzed after 6 hours, taking into consideration the initial increase in BTG. This observed stability could have positive logistical and economic implications, such as reducing the urgency for immediate sample processing and allowing for more flexible laboratory workflows. The presence of air in samples did not affect preanalytical stability in this study. Hemolysis remained minimal in EDTA samples throughout 48 h, whereas LDH, reflecting general cellular damage, showed a significant increase after 24 h, underscoring the importance of centrifuging blood and separating blood cells from plasma within 6 hours.

## Author Contributions

Benjamin S. Storm, Tom E. Mollnes, and Ole‐L. Brekke contributed to the conceptualization and design of the experiments. Benjamin S. Storm, Renathe H. Grønli, Kristin Pettersen, Åse E. Emblem, Ingrid S. Joakimsen, and Anne Landsem were responsible for conducting the experiments. Data analysis was performed by Benjamin S. Storm. Benjamin S. Storm and Ole‐L. Brekke drafted the manuscript.

## Funding

This research was funded by the Internal Research Fund at Nordland Hospital Trust, Norway (project number 11711‐2023).

## Disclosure

All authors critically revised and approved the final version for submission.

## Ethics Statement

The study was conducted using blood samples from healthy volunteers and was approved by the Regional Committee for Medical and Health Research Ethics (P REK Nord 32/2004).

## Consent

Written informed consent was obtained from all participants.

## Conflicts of Interest

The authors declare no conflicts of interest.

## Supporting Information

Additional supporting information can be found online in the Supporting Information section.

## Supporting information


**Supporting Information 1** Supporting Figure 1: Platelet activation in EDTA tubes during incubation estimated as β‐thromboglobulin and sP‐selectin release.

**Supporting Information 2** Supporting Figure 2: Hemolysis and cell death in EDTA tubes during incubation measured using spectrophotometry on the HemoCue low‐Hb and NanoDrop and by lactate dehydrogenase.

**Supporting Information 3** Supporting Figure 3: Blood gases and metabolites in citrate tubes.

**Supporting Information 4** Supporting Figure 4: Blood gases and metabolites in EDTA tubes.

**Supporting Information 5** Supporting Table 1: The calculated maximum acceptable bias of selected analytes.

## Data Availability

The data supporting the findings of this study are available from the corresponding author upon reasonable request.
