# Supplementary material for: Impact of Preanalytical Storage Time and Air Exposure on ROTEM and Coagulation Sample Stability
Source: Adv Hematol. 2026 Feb 9;2026:5145648. doi: 10.1155/ah/5145648 (PMC13508150; doi:10.1155/ah/5145648)

Supplementary Figure 1. Platelet activation.

Activation of platelets was assessed in blood incubated in Vacuette® EDTA tubes (n=6, three male, three female) by plasma β-thromboglobulin (BTG) (A) and sP-selectin (B) using ELISA. The tubes were filled using standard technique (white circles) or using standard technique and subsequently topped up with additional EDTA blood and capped, ensuring no air was present inside the tube (grey circles). Tubes were incubated upright for 48 hours at room temperature on a shaker running at 250 rpm. After 0, 2, 4, 6, 24 and 48 hours, a set of samples were analyzed. In panel A, the y-axis has a binary logarithmical scale. Horizontal lines are medians and error bars span the 25th to the 75th percentile. Changes from baseline in standard tubes and differences between standard tubes and full tubes were compared using RM-ANOVA with uncorrected Fischer's LSD test. Only significant ($p<0.05$) differences are shown on graphs.

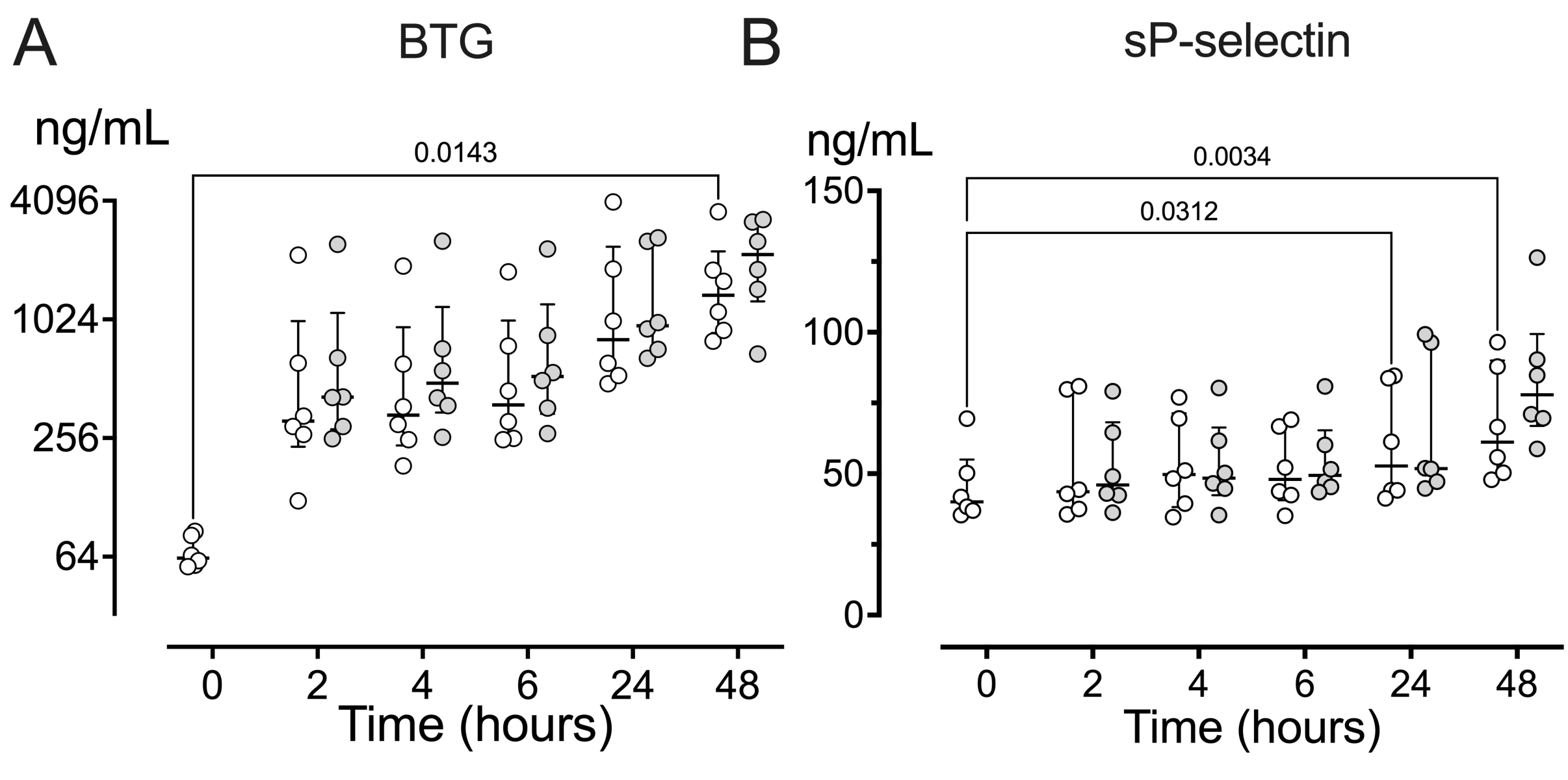

Supplement: Supplementary file 1 [file AH-2026-5145648-s001.pdf]
