# Supplementary material for: Impact of Preanalytical Storage Time and Air Exposure on ROTEM and Coagulation Sample Stability
Source: Adv Hematol. 2026 Feb 9;2026:5145648. doi: 10.1155/ah/5145648 (PMC13508150; doi:10.1155/ah/5145648)

Supplementary Figure 2. Hemolysis and cell death.

Hemolysis and cell death in blood incubated in Vacuette® EDTA tubes measured using spectrophotometry on the HemoCue® low-Hb dual wavelengths (570 and 880 nm) apparatus (A) and Nanodrop apparatus at 414 nm (B) and 600 nm (C) and by lactate dehydrogenase (LDH) on the Atellica® Clinical Chemistry Analyzer (D). White circles are standard tubes with air present in the tube. Grey circles are full tubes. Horizontal lines at the median. Error bars span the 25th to the 75th percentile. Only significant ($p<0.05$) differences are shown on the graphs.

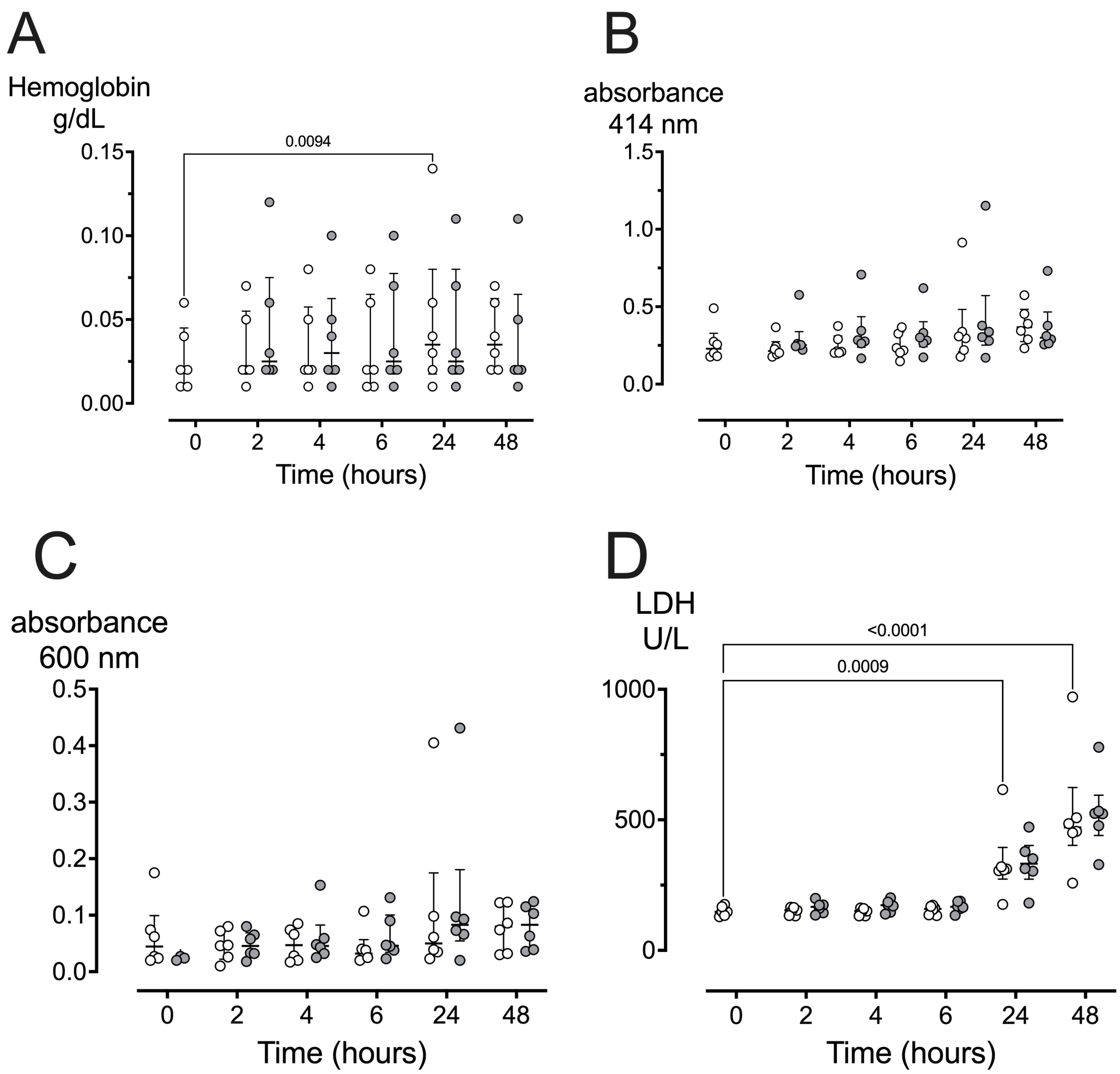

Supplement: Supplementary file 2 [file AH-2026-5145648-s002.pdf]
