# Supplementary material for: Impact of Preanalytical Storage Time and Air Exposure on ROTEM and Coagulation Sample Stability
Source: Adv Hematol. 2026 Feb 9;2026:5145648. doi: 10.1155/ah/5145648 (PMC13508150; doi:10.1155/ah/5145648)

Supplementary Figure 3. Blood gasses in citrated blood.

Blood gases and metabolites were measured in the blood (n=4, two male, two female) incubated in citrate Vacuette® tubes filled using standard technique (solid lines) and in tubes filled using standard technique and subsequently topped up with citrated blood and capped, ensuring no air was present inside the tube (dotted lines). Tubes were incubated upright for 48 hours at room temperature on a shaker running at 250 rpm. After 0, 2, 6, 24, and 48 hours, a set of blood gasses were analyzed using a Radiometer ABL800 Flex blood gas analyzer (Radiometer, Brønshøj, Denmark) as per manufacturer'sinstructions. Select results are shown: pH (panel A), pO_2 (panel B), pCO_2 (panel C), bicarbonate (panel D), hemoglobin (panel E), potassium (panel F), glucose (panel G), and lactate (panel H). Means are marked with dots. Error bars span the 95% CI.

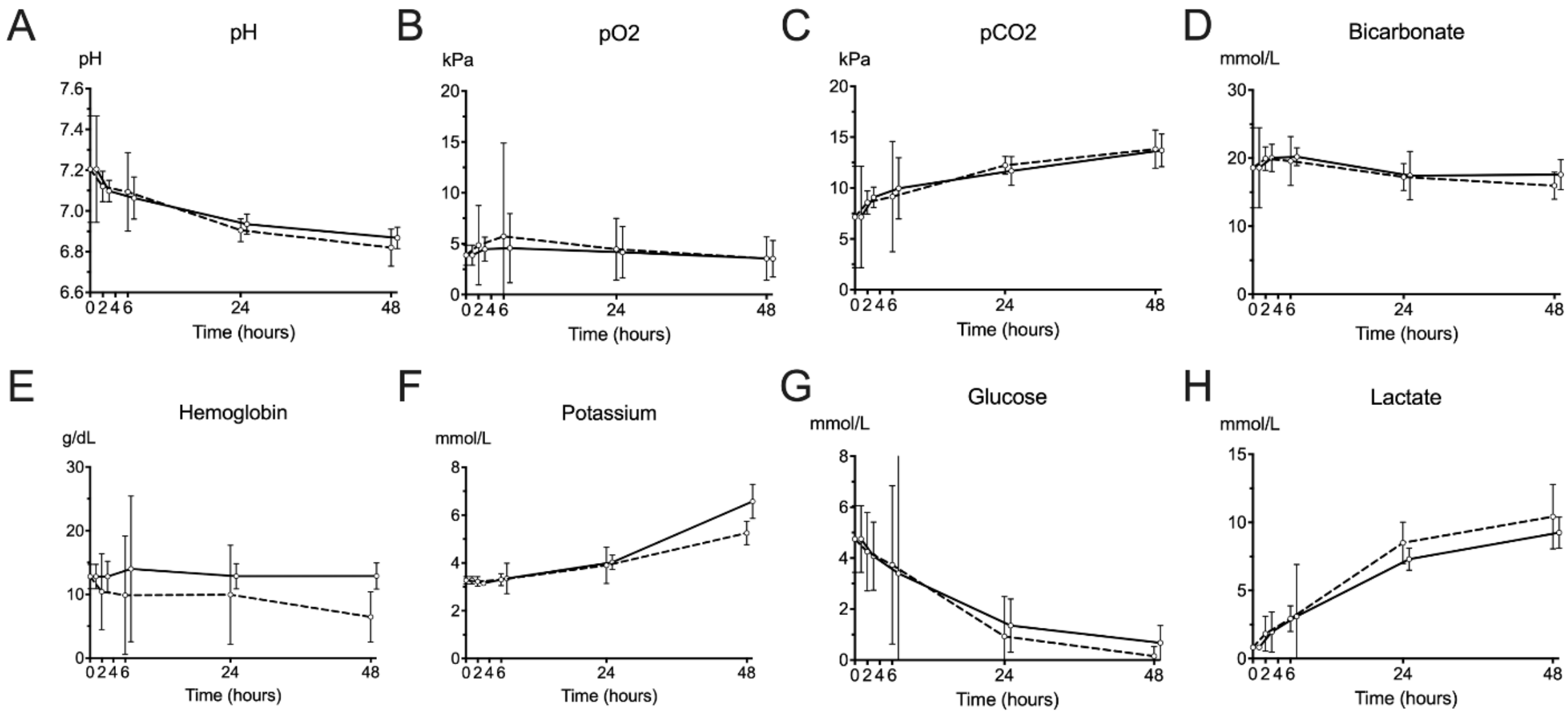

Supplement: Supplementary file 3 [file AH-2026-5145648-s003.pdf]
