# Supplementary material for: Impact of Preanalytical Storage Time and Air Exposure on ROTEM and Coagulation Sample Stability
Source: Adv Hematol. 2026 Feb 9;2026:5145648. doi: 10.1155/ah/5145648 (PMC13508150; doi:10.1155/ah/5145648)
Supplement: Supplementary file 5 [file AH-2026-5145648-s005.pdf]

**Supplementary Table 1. Maximum acceptable bias**

| Analyte | Maximum acceptable bias (%)[a] | Reference | EFLM data updated[b] |
|---|---|---|---|
| *Clinical chemistry* | | | |
| S-Potassium | 1.5 | EFML[c] | 08/11/2023 |
| Sodium | 0.3 | EFML | 14/12/2023 |
| Glucose | 2.4 | EFML | 14/12/2023 |
| Lactate | 9 | Diaz-Garzon Clin. Chim. Acta. (2021) 523. 497-503. | 14/12/2023 |
| Hemoglobin | 1.7 | EFML | 14/12/2023 |
| *Acid-base* | | | |
| Bicarbonate | 1.6 | EFML | 14/12/2023 |
| pCO2 | 1.8 | EFML | 14/12/2023 |
| pO2 | ±6 | LabQuality external quality controll, nov. 2023 | |
| pH | 1 | Philip Harding and Callul Fraser. Clinical Chemistry. 33/8. 1416-1418. 1987. | |
| Base excess | 22 | LabQuality External Quality control april 2024: ±2 SD | |
| *Coagulation* | | | |
| APTT | 1.9 | EFML | 14/12/2023 |
| INR | 1.3 | EFML | 14/12/2023 |
| BTG | No data | Guiseppe Banfi 2008. Seminars in | |
| PTF1+2 | No data | No reference | |
| sP-selectin | 11 | Graf et al. Clinical Biochemistry. 2017 (50). 942-946. | 24/01/2024 |
| *ROTEM parameters[d]* | | | |
| EXTEM CT | 6.7 | MB Mijovski et al. J. Cardiovasc. Dev. Dis. 2022, 9, 205 (with Rivaroxaban) | |
| EXTEM CFT | 6.2 | MB Mijovski et al. J. Cardiovasc. Dev. Dis. 2022, 9, 205 (with Rivaroxaban) | |
| EXTEM α-angle | No data | | |
| EXTEM MCF | 1.7 | MB Mijovski et al. J. Cardiovasc. Dev. Dis. 2022, 9, 205 (with Rivaroxaban) | |
| INTEM CT | 25 | External quality control UKNEQAS ROTEM Sigma, jul. 2022 | |
| INTEM CFT | 35 | External quality control UKNEQAS ROTEM Sigma, jul. 2022 | |
| INTEM α-angle | No data | | |
| INTEM MCF | No data | | |

Abbreviations: EFML European Federation of Laboratory Medicine database on biological variation. p partial pressure. INR international normalized ratio. APTT activated prothromboplastin time. BTG beta-thromboglobulin. PTF1+2 prothrombin fragment 1+2. ROTEM rotational thromboelastometry. CT clotting time. CFT clot formation time. MCF maximal clot formation. [a]Maximum acceptable bias is calculated as 0.375 x total biological variation. [b]Data update time from EFML webpage. [c]Aarsand AK, Sandberg S et al, EFML database on biological variation. Accessed 12/03/24. [d]Values specific to the ROTEM® delta machine.
